# Atypische Mykobakteriose der Haut

**DOI:** 10.1007/s00292-025-01442-w

**Published:** 2025-06-02

**Authors:** Anna Vetter, Hilte Geerdes-Fenge, Marco Saß, Maja Hühns, Anne-Sophie Becker

**Affiliations:** 1 Institut für Pathologie, Universitätsmedizin Rostock, Strempelstr. 14, 18057 Rostock, Deutschland; 2 Abteilung für Tropenmedizin und Infektionskrankheiten, Zentrum für Innere Medizin, Universitätsmedizin Rostock, Rostock, Deutschland; 3 Klinik für Unfall‑, Hand- und Wiederherstellungschirurgie, Chirurgische Klinik und Poliklinik, Universitätsmedizin Rostock, Rostock, Deutschland

**Keywords:** Granulomatöse Entzündung, Tuberkulose, Histologie, Differentialdiagnose, Molekularpathologie, Granulomatous inflammation, Tuberculosis, Histology, Differential diagnosis, Molecular pathology

## Abstract

Wir berichten über einen nicht immunsupprimierten 60-jährigen Patienten mit über mehrere Wochen progredienten, schmerzlosen subkutanen Noduli an der dominanten Hand bis Unterarm, die klinisch als Weichteiltumor vorrangig mesenchymaler Herkunft eingestuft wurden. Nach Exstirpation zweier Knötchen zur diagnostischen Abklärung zeigte die Histologie eine chronisch-granulomatöse Entzündung der Dermis und Subkutis mit zentralen, eosinophilen Nekrosen unter Einschluss von Zelldebris. Mittels Lichtmikroskopie wurden keine säurefesten Stäbchen nachgewiesen. Mittels molekularer Diagnostik konnte DNA von *Mycobacterium haemophilum* als nichttuberkuloides Mykobakterium (NTM) nachgewiesen werden. Diese Infektion als Ursache der Noduli konnte anhand der klassischen Histomorphologie einer nekrotisierend-granulomatösen Entzündung in Ergänzung mit einer PCR-basierten Erregerdiagnostik identifiziert werden. Dieses Fallbeispiel veranschaulicht die klinische Präsenz mykobakterieller Infektionen insbesondere extrapulmonaler und extranodaler Lokalisationen. Neben kutanen Infektionsherden können Infektionen durch NTM in Abhängigkeit von der Immunkompetenz des Wirts Multisystemerkrankungen unter Beteiligung des Gelenksystems verursachen, welche antibiotisch therapierbar sind.

## Abstract (en)

A 60-year-old immunocompetent patient presented with painless subcutaneous nodules on the right hand and forearm that progressed over several weeks. Nodules were clinically classified as a soft tissue tumor of primarily mesenchymal origin. After extirpation of two nodules for diagnostic clarification, histology showed a chronic granulomatous inflammation of the dermis and subcutis with central eosinophilic necrosis including cell debris. Light microscopy showed no acid-fast rods, but molecular diagnostics revealed DNA of *Mycobacterium haemophilum* as a nontuberculoid mycobacterium (NTM). The infection was identified as the cause of the nodules based on the classic histomorphology of necrotizing-granulomatous inflammation in addition to PCR-based pathogen diagnostics. This case illustrates the clinical presence of mycobacterial infections, especially in extrapulmonary and extranodal localizations. In addition to cutaneous foci of infection, NTM infections may cause multisystem disease involving the joint system, depending on the host’s immunocompetence, which can be treated with antibiotics.

## Anamnese

Ein 60-jähriger Akademiker stellte sich mit mehreren zunehmend größenprogredienten subkutanen Knötchen der Hand und des Unterarms rechts vor. Als einzige Vorerkrankung bestand ein bis dato nicht bekannter schwerer Vitamin-D-Mangel.

Die sonographisch maximal 12 mm messenden, derben, kaum verschieblichen Herde wurden ohne größere Schmerzsymptomatik als kosmetisch störend empfunden. Zu diagnostischen Zwecken wurden 2 der Knoten extirpiert und mit der klinischen Angabe „tastbarer Weichteiltumor Unterarm rechts mittig und Ellenbogen-nah“ formalinfixiert an die Pathologie übersandt.

## Histopathologischer Befund, molekulare Analytik und Diagnose

**Abb. 1:**
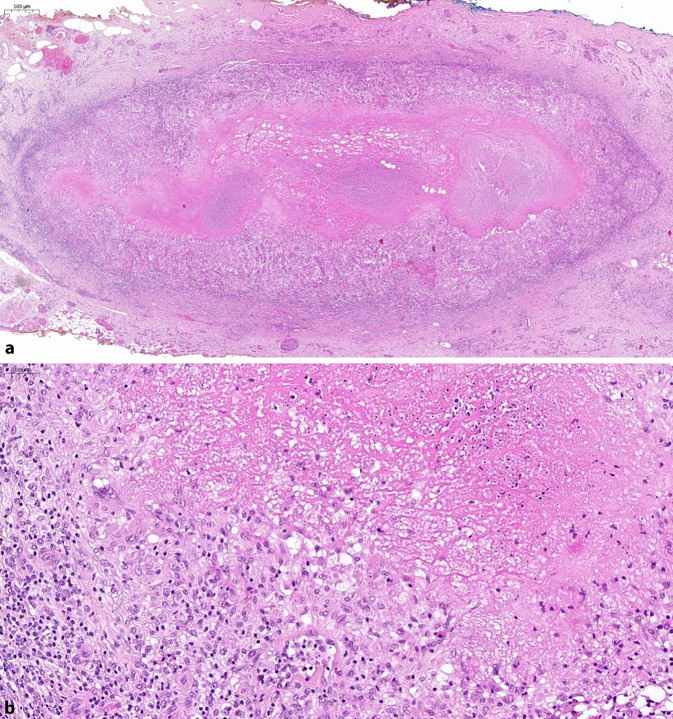
Repräsentative Histologie. **a** In der Übersicht eine umschriebene granulomatöse Entzündung im Unterhautweichgewebe mit zentraler eosiner Nekrose. Vergr. 20:1. **b** In hoher Vergrößerung hierum saumartig gelagerte Histiozyten und Epitheloidzellen sowie Fibroblasten und peripher gelegene Lymphozyten. Vergr. 200:1

**Abb. 2:**
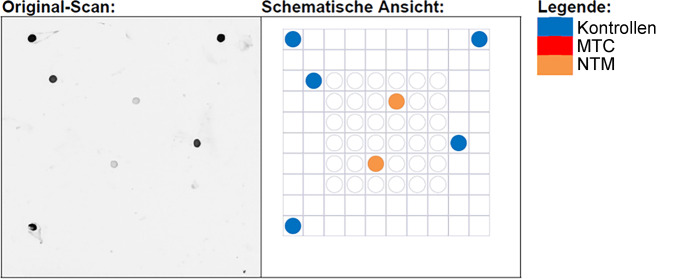
Hybridisierungsbasierte Visualisierung *M.* *haemophilum* spezifischer Gensequenzen. Nach Inkubation mit vorab aufgereinigter, amplifizierter DNA des formalinfixierten, in Paraffin eingebetteten Materials zeigen sich nach Hybridisierung auf dem Vision*Array*® MYCO Chip 2.0 (ZytoVision GmbH) 2 spezifische, *orange* Signale (*Pfeil*). Diese codieren DNA für das nichttuberkulöse Mykobakterium *M.* *haemophilum*. Die Intensität liegt über dem Schwellenwert, die 5 *blauen* internen Hybridisierungs- und Positivkontrollen weisen im Vergleich eine etwa 4-fach höhere Intensität auf (*links*: Originalscan, *rechts*: Schema). *MTC* Mycobacterium-tuberculosis-Komplex, *NTM* „non-tuberculoid mycobacteria“

Makroskopisch zeigten sich 2 je fadenmarkierte, bis zu 17 mm durchmessende Weichteilexzisate unter Einschluss zentraler, max. 10 mm messender grauer Knötchen. Mikroskopisch entsprachen diese einer chronischen Entzündungsreaktion mit zentral amorpher Nekrose umgeben von einem Randwall aus Fibroblasten, Lymphozyten, Histiozyten und Riesenzellen (Abb. 1). Mittels PAS- und Ziehl-Neelsen-Färbung gelang kein Erregernachweis innerhalb der Granulome, welche die tuberkeltypische verkäsende Morphologie aufwiesen. Nach Mikrodissektion und PCR-basierter Amplifikation entsprechender DNA-Sequenzen konnte mittels des Vision*Array*® MYCO Chip 2.0 (ZytoVision GmbH, Bremerhaven, Deutschland) [1] Nukleinsäure des nichttuberkulösen Mykobakteriums *M. haemophilum* nachgewiesen werden (Abb. 2). Somit ist als klinische Diagnose eine atypische Mykobakteriose der Haut durch *M. haemophilum *wahrscheinlich.

## Therapie und Verlauf

Da lediglich 2 kutane Herde entfernt wurden und eine Lymphadenopathie der rechten Axilla bestand, erfolgte unter der Annahme weiterer Infektherde eine systemische leitliniengerechte Therapie mit Moxifloxacin 400 mg, Azithromycin 500 mg und Rifampicin 600 mg für 6 Monate. Etwa 4 Wochen nach Therapiebeginn stellte sich der Patient mit Schwellungen an beiden Narben und erhöhter Temperatur wieder vor. Nach Ausschluss einer Wundinfektion bzw. eines Seroms bestand der Verdacht eines Immunrekonstitutionssyndrom-Inflammations-Syndroms als paradoxe Reaktion auf die antibiotische Behandlung. Daraufhin wurde ein Therapieversuch mit Prednisolon (40 mg täglich per os) begonnen, was zu einer Befundbesserung führte und in der Fortführung der Prednisolontherapie für 5 Tage mit abschließendem Ausschleichen resultierte.

Der Patient berichtete 10 Monate nach Therapiebeginn über eine vollständige Abschwellung der Noduli, welche auch nach Absetzen der antimykobakteriellen Therapie nicht erneut auftraten. Jenseits einer Vitamin-D-Substitution (20.000 IE wöchentlich) besteht aktuell keine weitere Therapie.

## Diskussion

Die granulomatöse Entzündung ist eine Differentialdiagnose für kutane Raumforderungen [2, 3]. Die Morphologie der Granulome gibt unter Berücksichtigung der Anatomie (Lymphknoten *versus* extranodale Lokalisation) Hinweise für die auslösende Erkrankung [4]. Begleitende Nekrosen finden sich beispielsweise in rheumatoiden Granulomen oder Granulomen infektiöser Genese. Sogenannte verkäsende Granulome sind typisch für eine Infektion mit Mykobakterien. Diese Gattung inkludiert neben *M. tuberculosis *auch *M. leprae *und *nichttuberkulöse Mykobakterien* (NTM), wozu *M. haemophilum *kategorisiert wird. Die Infektion mit diesem säurefesten Stäbchen induzierte in der vorgestellten Kasuistik eine knötchenförmige Entzündungsreaktion. Allgemein grenzt eine solche erregerbedingte granulomatöse Entzündungsreaktion den Keim durch ein dichtes Infiltrat aus Entzündungszellen ab, ohne ihn durch Phagozytose oder Zytotoxizität eliminieren zu können. In frühen Stadien kann das histologische Bild zudem durch neutrophilenreiche/abszedierende Infiltrate (suppurative Granulome) geprägt sein (Tab. 1).

**Tab. 1:** Stadieneinteilung kutaner Infektionen mit *Mycobacterium haemophilum*

Stadium	Definition/klinische Präsentation
1: isolierter kutaner Befall	Einzelne, anatomisch umschriebene kutane Manifestationen
2: disseminierter kutaner Befall	Multiple kutane Manifestationen ohne weiteren Organbefall
3: disseminierter Befall	Manifestation an Haut und weiteren (inneren) Organen, typischerweise Gelenk/Knochen in Form einer Arthritis/Osteomyelitis

Infektionen mit *M. haemophilum *zeigen eine steigende Inzidenz [5, 6]. Neben sensitiveren Untersuchungsmethoden zur Detektion des Erregers, dessen Nachweis mittels Ziehl-Neelsen-Färbung hier nicht gelang, ist diese Häufung auch durch eine zunehmende iatrogene Immunsuppression zu erklären: PatientInnen mittleren Alters mit Einnahme von Glucocorticoiden, Mycophenolat-Mofetil, Ciclosporin und Cyclophosphamid scheinen besonders anfällig für Infektionen mit *M. haemophilum* zu sein. Die Prognose ist dabei unter Verwendung einer Dreifachmedikation aus Chinolon, Makroliden und Rifampicin in Stadien mit kutanem Befall exzellent. Als anzunehmende Prädisposition des hier vorgestellten Patienten für die Infektion konnte eine Hypovitaminose D identifiziert werden [7]. Der Ort der Erstinfektion mit dem Bakterium konnte ebenso wie die Eintrittspforte nicht sicher eruiert werden.

## Fazit für die Praxis

- Infektionen mit nichttuberkulösen Mykobakterien präsentieren sich klinisch vielfältig und können neoplasieverdächtige Gewebeschwellungen imitieren.
- Die gewebebasierte Diagnostik am formalinfixierten, in Paraffin eingebetteten Material kann mykobakterielle DNA-Sequenzen mittels molekularpathologischer Methoden nachweisen.
- Neben *M. tuberculosis* können in Abhängigkeit von der angewandten Technik weitere nichttuberkulöse Mykobakterien nachgewiesen und in Folge entsprechend antibiotisch therapiert werden.
- Bei unklaren Raumforderungen mit dem Bild einer nekrotisierenden granulomatösen Entzündung kann zusätzlich zur histologischen Untersuchung eine mikrobiologische Untersuchung auf Mykobakterien durchgeführt werden.
